# Mesenchymal Stem Cell-Based Treatment for Microvascular and Secondary Complications of Diabetes Mellitus

**DOI:** 10.3389/fendo.2014.00086

**Published:** 2014-06-06

**Authors:** Grace C. Davey, Swapnil B. Patil, Aonghus O’Loughlin, Timothy O’Brien

**Affiliations:** ^1^Regenerative Medicine Institute (REMEDI) and Biosciences Building, National University of Ireland, Galway, Ireland; ^2^Department of Medicine, Galway University Hospital (GUH), Galway, Ireland

**Keywords:** mesenchymal stromal cell, MSC, diabetes, microvascular complication, retinopathy, nephropathy, neuropathy

## Abstract

The worldwide increase in the prevalence of Diabetes mellitus (DM) has highlighted the need for increased research efforts into treatment options for both the disease itself and its associated complications. In recent years, mesenchymal stromal cells (MSCs) have been highlighted as a new emerging regenerative therapy due to their multipotency but also due to their paracrine secretion of angiogenic factors, cytokines, and immunomodulatory substances. This review focuses on the potential use of MSCs as a regenerative medicine in microvascular and secondary complications of DM and will discuss the challenges and future prospects of MSCs as a regenerative therapy in this field. MSCs are believed to have an important role in tissue repair. Evidence in recent years has demonstrated that MSCs have potent immunomodulatory functions resulting in active suppression of various components of the host immune response. MSCs may also have glucose lowering properties providing another attractive and unique feature of this therapeutic approach. Through a combination of the above characteristics, MSCs have been shown to exert beneficial effects in pre-clinical models of diabetic complications prompting initial clinical studies in diabetic wound healing and nephropathy. Challenges that remain in the clinical translation of MSC therapy include issues of MSC heterogeneity, optimal mode of cell delivery, homing of these cells to tissues of interest with high efficiency, clinically meaningful engraftment, and challenges with cell manufacture. An issue of added importance is whether an autologous or allogeneic approach will be used. In summary, MSC administration has significant potential in the treatment of diabetic microvascular and secondary complications but challenges remain in terms of engraftment, persistence, tissue targeting, and cell manufacture

## Introduction

Diabetes mellitus (DM) is a heterogeneous group of metabolic disorders characterized by hyperglycemia with impaired metabolism of carbohydrate, fat, and proteins as a result of defects in insulin secretion, insulin action, or both (1). Diabetes is one of the world’s oldest diseases and it has been centuries since this syndrome was first recognized (2, 3). Considered previously as a disease of the affluent, diabetes is now considered an epidemic. The world prevalence of DM in 2010 was estimated at 6.4% or 285 million adults and it is expected this number will rise to 439 million adults in 2030. Most of this increase will occur in developing countries (4).

Type 1 DM (T1DM) is caused by β-cell destruction. The pathogenesis of T1DM is the autoimmune destruction of the pancreatic β-cells that leads to loss of insulin secretion and absolute insulin deficiency. Type 2 DM (T2DM) is caused by a combination of genetic and non-genetic factors that result in insulin resistance and insulin deficiency. Non-genetic factors include increasing age, high caloric intake, obesity, central adiposity, sedentary lifestyle, and low birth weight. This group comprises approximately 90–95% of cases in the diabetes syndrome (5). Chronic hyperglycemia in diabetes leads to various metabolic, hormonal, and physiologic alterations in the body, which further develop a number of secondary complications, which are responsible for major morbidity and mortality (6).

These complications are wide ranging and are grouped into “macrovascular complications” and “microvascular complications.” Macrovascular complications arise due to chronic hyperglycemia and associated cardiovascular risk factors affecting the arteries that supply the heart, brain, and lower extremities. The major macrovascular complications include accelerated cardiovascular disease (CVD) resulting in higher risk of coronary artery disease (CAD), peripheral arterial disease, myocardial infarction (MI), stroke, and limb amputation (7, 8). The complications resulting from damage to small blood vessels are grouped as microvascular complications. Persistent chronic hyperglycemia resulting in development of diabetes-specific microvascular complications in the retina, renal glomerulus, and peripheral nerves are characteristic of all forms of diabetes. As a consequence of its microvascular pathology, diabetes is the leading cause of blindness, end-stage renal disease, and a variety of debilitating neuropathies (7). Microvascular complications are classified into retinopathy, nephropathy, and neuropathy. Other secondary complications associated with diabetes include diabetic foot ulcers (9), cardiomyopathy (10), depression (11), erectile dysfunction (12), increased fracture risk and impaired bone healing (13), and cutaneous manifestations (14). In this review, microvascular complications such as retinopathy, nephropathy, and neuropathy are discussed and the application of mesenchymal stromal cell therapy to the treatment of multiple diabetic complications is examined in detail. While each diabetic complication tends to be considered in isolation, the EU Commission have recently funded a consortium, REDDSTAR, which will focus on the use of mesenchymal stromal cells (MSCs) in the treatment of all microvascular complications of DM.

## Diabetic Microvascular Complications

### Diabetic retinopathy

DM is associated with development of several ocular complications and diabetic retinopathy (DR) is the most severe among these ocular complications (15). It is the most frequent cause of new cases of blindness among adults aged 20–74 years (16). As compared to T2DM patients, individuals with T1DM are at higher risk for development of more severe retinal complications and visual loss. However, T2DM patients account for approximately 90% of the population with DM, and they comprise a larger proportion of those affected with DR (17). Clinically, there does not seen to be differences in symptoms in type 1 or type 2 DM and nearly all patients with long-term (20 years) DM will show some retinal lesions (20, 18).

Diabetic retinopathy is clinically classified into non-proliferative and proliferative disease stages. In non-proliferative diabetic retinopathy (NPDR), there are only intraretinal microvascular changes. The abnormalities due to non-proliferative retinopathy include microaneurysms, small outpouchings from retinal capillaries, altered retinal vascular permeability leading to intraretinal abnormalities, and eventual retinal vessel closure. Retinal vessel closure leads to non-perfusion, seen clinically as increasing dot intraretinal hemorrhages, venous abnormalities, and intraretinal microvascular abnormalities (15, 17). In this initial stage of NPDR, most people do not notice any visual impairment (8).

Proliferative diabetic retinopathy (PDR) involves the formation of new blood vessels on the retina or the optic disk. These new abnormal blood vessels erupt through the surface of the retina and proliferate into the vitreous cavity of the eye and can hemorrhage into the vitreous, resulting in visual loss. Late in the course of the disease, new blood vessels may form within the stroma of the iris and may extend, with formation of fibrosis, into the structures that drain the anterior chamber angle of the eye (15). Hyperglycemia (20), hypertension (21), and dyslipidemia are considered as the major risk factors for DR (22). Intensive glycemia control (16, 23) and maintenance of blood pressure have greatly reduced the risk of blindness from this disease, but retinopathy remains an important complication in diabetic patients. In addition to glycemic control and blood pressure maintenance, other treatments include injection of the steroid triamcinolone, and more recently, vascular endothelial growth factor (VEGF) antagonists into the eye (8), laser photocoagulation, and vitrectomy. These treatments are helpful in reducing the vision loss but are invasive in manner and can lead to loss of visual field, visual acuity loss, or risk of severe postoperative visual loss.

### Diabetic nephropathy

Diabetic nephropathy is one of the leading causes of end-stage renal failure in the western world (24). It is the major cause of morbidity and mortality in T1DM patients and is becoming a serious clinical problem in T2DM patients (25). After presentation, the initial changes in kidney include increased renal blood flow, hypertrophy, glomerular hyperfiltration, and hyperperfusion. These early stage changes are reversible and are not considered as a reliable indicator for the development of diabetic nephropathy (26, 27). Persistent hyperglycemia for several years further induces structural and cellular effects in the kidney. The significant structural changes includes thickening of the glomerular basement membrane, glomerular hypertrophy, increased extracellular matrix accumulation (tubulointerstitial fibrosis) with mesangial expansion, and modest expansion of the tubulointerstitium (25, 26, 28). High blood glucose induces cellular changes in various types of cells present in the kidney. The major cellular abnormalities develop in glomerular epithelial cells (podocytes), which includes broadening of podocyte foot processes with progressive decrease in their number and density per glomerulus (29, 30).

Clinically, there is a decline in glomerular-filtration rate (GFR) with progressive increase in urinary albumin excretion, and in association with an increase in blood pressure, it ultimately leads to end-stage renal failure (25). The earliest manifestation of diabetic kidney disease can be detected by the presence of microalbuminuria, a state known as incipient diabetic nephropathy, where there is presence of small amounts of albumin in the urine (30–300 mg/day) (31). Microalbuminuria is considered as the earliest marker and predictive of the development of proteinuria or overt nephropathy, particularly in T1DM (25). It represents a potentially reversible state of nephropathy and is sought in diabetic management programs as a matter of routine. After the phase of microalbuminuria, there is a continued increase in urinary protein excretion with declining GFR. This results in the development of Albustix-positive proteinuria and is known as overt nephropathy or macroproteinuria. In diabetic patients with more than 5 years of hyperglycemia, appearance of persistent albuminuria [albumin excretion rate (AER) > 300 mg/24 h] without any urinary tract infection (UTI), other renal diseases or heart diseases represents diabetic nephropathy (32). If left untreated, uremia will supervene and require referral to end-stage renal failure programs, such as dialysis or transplantation.

Hypertension plays a critical role in the progression of diabetic nephropathy. Controlling the blood pressure shows significant renoprotective and antiproteinuric effects. In addition, lowering blood pressure reduces albuminuria and attenuates the rate of loss of GFR in both T1DM and T2DM patients (30, 33). The major strategies currently used to reduce the risk of onset or progressions of diabetic nephropathy are glycemic control along with intensive management of systemic blood pressure. The major strategy used to manage blood pressure is modification in renin–angiotensin system (RAS) by means of angiotensin-converting enzyme (ACE) inhibitors and/or angiotensin II (ANG II) receptor antagonists (34, 35).

### Diabetic neuropathy

Diabetic peripheral neuropathy is one of the most frequent complications of DM. Sixty-six percentage of people with T1DM and 59% of people with T2DM have objective evidence of peripheral neuropathy (36). The pathophysiology of diabetic neuropathy (DN) remains complex and not fully elucidated. The consequences of DN include impaired quality of life, pain, foot deformity, neuropathic ulceration, and amputation.

The pivotal DCCT (Diabetes Control and Complications Trial) supports the hypothesis that DN occurs as a result of high glucose concentrations, however, subsequent research has implicated several more biological mechanisms in the pathogenesis of DN (37). Hyperglycemia results in oxidative stress and reactive oxygen species (ROS) generation in addition to advance glycation end product (AGE) production. This results in sensory, motor, and autonomic nerve dysfunction (37). Vascular insufficiency, ischemia, hypoxia, dyslipidemia, metabolic syndrome, and impaired insulin signaling are implicated in the development and progression of DN (37). Pro-inflammatory cytokines contribute to the pathogenesis of neuropathy and neuropathic pain. Injury to peripheral nerves results from the production of cytokines that originate from resident and recruited lymphocytes, macrophages, neurons, and Schwann cells. Patients with both T1DM and T2DM exhibit elevated blood levels of tumor necrosis factor-α (TNF-α), and medications, which bind TNF-α improves nerve conduction velocity in rodents (37). The inflammatory cytokines interleukin-2 and interleukin-6 are also increased as a result of hyperglycemia, with patients suffering from painful DN displaying an increased level of high sensitivity C-reactive protein as compared to non-painful DN (38, 39).

Diabetic neuropathy is characterized by a progressive neuronal loss, dymyelination, and impaired nerve regeneration with ultimately dysfunction of nerve fibers affecting both the autonomic and somatic divisions of the nervous system (39). Neuropathic ulceration, painful neuropathy, and autonomic dysfunction are the consequence of DN. Distal symmetrical polyneuropathy is the primary cause of plantar ulceration. Nerve damage involves sensory, motor, and autonomic nerves and subsequently the patient’s ability to perceive pain, pressure, touch, and temperature is altered (40). Motor neuropathy affects the small muscles of the foot and causes weakness, atrophy, and deformity. The deformities include clawing of the toes, prominent metatarsal heads with increased plantar pressure and limited joint mobility. Autonomic neuropathy may reduce sweating and increase the temperature of the foot, predisposing to infection, and ulceration. The reduction in sweating and increased temperature predispose to cracking of the skin and consequent ulceration. Charcot’s neuroarthropathy is the result of bony dislocation and collapse of the arch. Autonomic dysfunction is implicated with abnormal perfusion to foot bones. The “rocker-bottom” deformity is prone to increased pressure and ulceration (40).

Persistent neuropathic pain interferes significantly with quality of life, impairing sleep, and emotional well-being, and is a significant causative factor for anxiety, loss of sleep, and non-compliance with treatment. There is evidence suggesting an association between neuropathic pain and depression, as for other types of pain (41). A causative link has also been suggested between DN and DR. In a rodent model of T2DM, it was shown that the decrease in endothelial progenitor cell (EPC) release from diabetic bone marrow was caused by bone marrow neuropathy and that these changes preceded the development of DR (42). The cornerstone of treatment of neuropathy is optimization of glycemic control, thus improving symptoms and preventing progression of DN. Currently available pharmacological therapies are frequently ineffective and are associated with multiple side-effects. The first-line symptomatic treatments, which benefit DN include antidepressants, e.g., serotonin–noradrenaline reuptake inhibitors (e.g., duloxetine), tricyclic antidepressants (e.g., amitryptyline) and anticonvulsants (e.g., gabapentin and pregabalin), and topical lidocaine in addition to topical capsaicin cream provide symptomatic relief. The addition of opiod analgesia may benefit in refractory cases of painful DN (41, 43) and α-lipoeic acid has demonstrated benefit in treatment of painful neuropathy (43).

Diabetic neuropathy results in an increased risk of foot ulceration. When patients present with a diabetic foot ulcer, assessment of vascular supply is critical. If vascular supply is intact these ulcers frequently represent neuropathic ulcers, and the treatment includes debridement and off weight bearing. Non-healing ulcers may be associated with infection and risk of amputation. If the etiology of the ulcer is ischemic, blood flow will need to be restored, or amputation may be necessary.

## Mesenchymal Stromal Cells

The discovery and characterization of stem cells and their innate properties has highlighted their potential as therapeutic agents in regenerative medicine, in particular for the treatment of cardiovascular, musculoskeletal, neurodegenerative, and immunological disorders, which heretofore have achieved only modest success rates. Stem cells can be broadly characterized by their source and tissue, they are typically generated from, and their differentiation capacity *in vitro*. The current manuscript will focus on the potential use of MSCs in the treatment of microvascular and secondary complications of DM.

### MSC classification

Mesenchymal stromal cells have been highlighted as a new emerging regenerative therapy in recent years. MSCs are progenitors of all connective tissue cells and the International Society for Cellular Therapy has defined three minimum requirements for classification of cells as MSC; they must be plastic adherent in normal culture conditions, differentiate into osteoblasts, adipocytes, and chondroblasts *in vitro* and express a defined population of cell surface markers (44). MSCs have the capacity of self-renewal and are multipotent, having the potential to differentiate into multiple cell types such as adipocytes, chondrocytes, and osteoblasts, but also differentiation into myocytes and neurons has been proposed (45–49). They can be derived from many different organs and tissues such as bone marrow, adipose tissue, nervous tissue, amniotic fluid, umbilical cord, placenta, menstrual blood, and dental pulps (50–53). MSCs are a subset of cells that express on their surface CD54/CD102, CD166, CD49 as well as CD73 and CD90. They also express CD44, CD105, whereas they do not express CD34, CD14, CD45, CD11a/LFA-1, and CD31, which are surface markers of hematopoietic cells and/or endothelial cells (44, 54). Although their differentiation capacity is less than other cell types such as embryonic stem cells (ESC) or induced pluripotent stem cells (iPSC), they still hold great promise for clinical applications having been demonstrated to play a role in tissue repair and regeneration in both pre-clinical and clinical studies, as they are able to migrate and home to injured sites, where they act both by regenerating tissues and by secreting trophic factors and paracrine mediators. They also have remarkable immunosuppressive properties secreting cytokines and immunomodulatory substances, and it is this property that has received most attention in recent years (55–60).

### MSCs in tissue repair

Mesenchymal stromal cells are believed to have an important role in tissue repair (56). Upon tissue injury immune/inflammatory cells, such as macrophages, neutrophils, CD4^+^ and CD8^+^ T cells, and B cells are activated by factors from damaged cells and vessels, and inflammatory molecules such as TNF α, IL-1β, free radicals, and chemokines are released by phagocytes in response to damaged cells and tissue. These immune cells and inflammatory molecules together with fibroblasts and endothelial cells are responsible for changes in the micro environment of the damaged tissue that results in the recruitment and differentiation of MSCs that can replace damaged tissue cells (61–63). In addition, many factors including TNF-α, IL-1, IFN-γ, and hypoxia can stimulate the release of growth factors from MSCs, such as epidermal growth factor (EGF), fibroblast growth factor (FGF), platelet-derived growth factor (PDGF), transforming growth factor-β (TGF-β), insulin growth factor-1 (IGF-1), and angiopoietin-1 (Ang-1), among others (64–67). These growth factors, in turn, promote the development of fibroblasts, endothelial cells, and tissue progenitor cells, which carry out tissue regeneration and repair.

### MSCs as immune modulators

Evidence in recent years has demonstrated that in addition to their differentiation capacity and involvement in tissue repair, MSCs have potent immunomodulatory functions. Through production of soluble factors, MSCs can alter the secretion profile of dendritic cells (DCs) resulting in increased production of IL-10, an anti-inflammatory cytokine, and decreased production of IFN-γ and IL-12. MSCs can inhibit T cell production and increase the number of CD4^+^CD25^+^FoxP3^+^ T-regulatory cells that suppress the immune response (68, 69). MSCs can inhibit proliferation and IgG secretion of B cells (70). Recent studies have shown that un-stimulated MSCs are indeed incapable of immunosuppression; they become potently immunosuppressive upon stimulation with the supernatant of activated lymphocytes, or with combinations of IFN-γ with TNF-α, IL-1α, or IL-1β. This observation revealed that under certain circumstances, inflammatory cytokines can actually become immunosuppressive (56). However, other studies have demonstrated that MSCs can be recognized by the host immune system. In some experimental conditions, MSCs infused into allogeneic, MHC-mismatched mice have been rejected (71, 72). Still their unique immunomodulatory properties made these cells appropriate for both autologous and allogeneic transplant investigations, as historically they have been considered poorly immunogenic. For the same reason, they have been proposed as a treatment for autoimmune diseases (73), and have been used for the treatment of experimental models of rheumatoid arthritis (RA), systemic lupus erythematosus (SLE), multiple sclerosis (MS), and DM with its associated complications (74–78).

More recently, the immune privileged status of MSCs has been questioned. Intracardiac allogeneic porcine MSCs elicit an immune response despite their low immunogenic profile *in vitro* (79). IV injection of allogeneic MSCs in rats lead to the formation of alloantibodies with the capacity to facilitate complement mediated lysis, and this allo-MSC induce immune response was sufficient to significantly reduce survival of subsequently injected allogeneic MSCs (80). A recent review of the published literature in the area indicated that the majority of studies documented some cellular and humoral immune responses against donor antigens following administration of non-manipulated, interferon activated, and differentiated allo-MSCs and the authors recommended the anti-donor immune responses elicited by allo-MSCs be studied in more detail (81).

### MSCs and glycemic control in DM

An attractive feature of MSCs in the treatment of diabetic microvascular complications is the reported ability of this cell type to improve glycemic control, which can subsequently benefit microvascular complications through systemic effects. Thus, it is conceivable that MSCs can simultaneously treat hyperglycemia and have a trophic effect on the underlying diabetic microvascular complications. A variety of preclinical animal models have shown a beneficial effect of MSC transplantation on glycemia through a direct effect of differentiation to cells capable of producing insulin (less likely), or an indirect effect of secretion of immunomodulators, which prevent endogenous T cells from eliciting pancreatic β-cell destruction, or other as yet unknown factors, which influence insulin secretion or action. Initial studies demonstrated that bone marrow cells had the capacity to differentiate into insulin-producing cells, and aggregates of these cells when transplanted into the kidney capsule of diabetic mice could lower blood glucose and maintain near normal glucose levels for 90 days. Graft removal resulted in rapid relapse and death of the experimental animals (82). In an animal model of T1DM where rodents were induced to develop diabetes through streptozotocin (STZ), MSC were able to differentiate into insulin-producing cells, releasing insulin in a glucose dependant manner and improving diabetic symptoms (83, 84). These insulin-producing cells express multiple genes related to the development or function of pancreatic beta cells, including high expression of pancreatic and duodenal homeobox 1, insulin, and glucagon (84) and were able to release insulin in a glucose-dependent manner that led to amelioration of diabetic conditions in STZ-treated nude mice (84, 85). Transplantation of undifferentiated MSCs into STZ-induced diabetes in C57Bl/6 mice induced normoglycemia and reversed glycosuria. This was accompanied by improved renal function and histological evidence of regeneration of normal beta pancreatic islets (86). In non-obese diabetic NOD mice, the injection of MSC reduced the capacity of diabetogenic T cells to infiltrate pancreatic islets thus preventing β-cell destruction (87). An additional cooperative action of MSC’s on co-transplantation with pancreatic islets results in improved graft morphology and improved revascularization indicating that possible trophic factors secreted by MSCs are aiding islet engraftment (88). Furthermore, multiple IV infusions of MSCs to a rodent model of T2DM resulted in normalization of blood glucose levels, which remained stable for 9 weeks after infusion. Serum concentrations of insulin and C-peptide were dramatically increased after MSC infusion and damaged pancreatic islets were restored to near normal with the ratio of insulin-positive cells per islet achieving near normal levels (89).

## MSC-Based Therapies for Diabetic Microvascular Complications

The worldwide increase in the prevalence of DM has highlighted the need for increased research efforts into treatment options for both the disease itself and its associated complications. Of particular concern is the increasing prevalence of diabetes affecting adolescents and young adults (90) promoting an earlier development of chronic illness caused by hyperglycemia, which is characterized by microvascular complications (retinopathy, nephropathy, and neuropathy) prolonged/incomplete wound healing, cardiomyopathy, or impaired bone repair. Both T1DM and T2DM patients can develop secondary complications, the risk of which is related to the duration of diabetes and the degree of glycemic control (91). This has prompted investigators not only to analyze the effect of stem cells and in particular MSC transplantation on glycemic control but also to assess the beneficial effects of MSCs on the resultant secondary diabetic complications.

### MSC-based therapies for diabetic retinopathy

A variety of animal models have been used to investigate DR (92) and these species do develop at least the early stages of retinopathy, including retinal capillary degeneration. The severity of disease in these models does increase with diabetes duration, however symptoms are still considered mild compared to long-term DR symptoms seen in patients, partly due to the limited lifespan of these laboratory animal models. Thus, the current animal models of DR are not considered fully reflective of human DR (93). Investigators have turned their attention to non-diabetic models of retinal neovascularization caused by branch vein occlusion, oxygen-induced retinopathy (94), or overexpression of growth factors such as VEGF (95) or IGF-1 (96). Most studies investigating cell effects of DR have involved EPC and those patients with earlier stages of disease (NPDR) exhibit reduced number of EPC compared to higher levels in patients with PDR (97, 98), most probably due to inflammatory reactions in damaged tissue and increased mobilization of EPC from the bone marrow in later stages of disease.

To date, there are no clinical studies evaluating the effect of adult stem cells in DR, however, animal models do give an indication of possible mechanisms of vascular repair. In studies carried out on ischemic retinal injury, adult stem cells (hematopoietic/mesenchymal) participate in retinal repair, homing into damaged areas, and differentiating into endothelial cells, microglia, and astrocytes (99–102). It has been shown that bone marrow-derived MSCs can differentiate into retinal cells and endothelial cells and rescue photoreceptors in the diseased retina (100, 103, 104). Animal studies have demonstrated that subretinal transplantation of MSCs delays retinal degeneration and preserves retinal function through a trophic response (105). In a rat model of retinitis pigmentosa, IV administration of BM MSCs prevented photoreceptor loss and preserved visual function (106). In an STZ rodent model of DR, intravenous injection of adipose-derived MSC resulted in a significant reduction in blood glucose levels in treated rats 1 week after transplantation, an improvement in the integrity of the blood–retinal barrier and evidence of the presence of donor cells in the retinas of treated rats and differentiation into photoreceptor cells and astrocytes (101). It has also been demonstrated that a single intravitreal injection of placental derived MSCs results in a significant decrease in retinal apoptosis in rodents rendered diabetic by a single STZ injection, by virtue of increased intravitreal, and retinal concentrations of neuroprotective growth factors (107).

However, there are questions as to the therapeutic value of angiogenic cell-based strategies in long-term PDR as they may risk worsening the aberrant reactive neovascularization in PDR that follows ischemic retinal injuries. To this end, some investigators have suggested a combination therapeutic approach of MSC administration with the HMG-CoA reductase inhibitor atorvastatin, which may prevent excess VEGF production by MSCs under hypoxic conditions and have the potential to improve viability and homing of the transplanted MSCs (108).

### MSC-based therapies for diabetic nephropathy

Animal studies have indicated that MSCs are successful candidate cell therapies for both the prevention and treatment of diabetic nephropathy. In studies where immune compromised NOD/SCID mice received systemic administration of human MSC’s or C57Bl/6 mice received murine MSC’s after induction of diabetes by STZ, results indicated that hyperglycemia was reversed and nephropathy was either prevented or repaired (78, 86). The result of systemic administration of MSCs in these studies was a reduction in blood glucose with an associated improvement of kidney function. After an intracardiac infusion of MSC, 11% of these cells engrafted into the kidneys, where they differentiated into endothelial cells. The engraftment of hMSCs into kidney was associated with improvements in glomerular morphology, a decrease in mesangial thickening, and a decrease in macrophage infiltration (78). It is important to confirm whether these beneficial effects on nephropathy were due to improvement in glycemia or a direct effect on the kidney. MSCs also had the ability to slow the progression of diabetic nephropathy through mechanisms independent from glycemic control as in a subsequent study comparing MSC-treated versus untreated DM mice, MSC-treated mice remained hyperglycemic but exhibited basal levels of albuminuria and very minor tubular dilation (109).

The effect of MSC administration on glucose levels and kidney function has also been assessed in other rodent models. In studies where diabetes was induced in Sprague-Dawley rats by a single IP injection of STZ, introcardiac infusion of bone marrow MSC resulted in a decrease in blood glucose, Alb/Cr ratio and renal mass index compared to control (110). The renoprotective effect of human umbilical cord derived MSCs has been examined in a rodent model of DM. Four weeks after STZ injection, hUMB cord derived MSCs prevented diabetic renal injury (except renal and glomerular hypertrophy) without a significant effect on blood glucose (111, 112) indicating that paracrine factors may be involved in the protection. The injection of hUMB cord MSCs after induction of hyperglycemia effectively prevented proteinuria and increased fractional mesangial area albeit with a low level of kidney engraftment (112). Autologous transplantation of adipose-derived MSCs minimized pathological alterations, reduced oxidative damage and suppressed the renal expression of pro-inflammatory cytokines in a rodent STZ model of diabetic nephropathy (113). Adipose MSC implantation significantly alleviated all indices of metabolic dysfunction when compared to controls including a reduction in blood glucose. Expression of MAPK signaling pathway molecules (p-p38, p-ERK and p-JNK) was also reduced in MSC-treated rodent renal tissues leading the authors to suggest that the positive therapeutic effect of adipose-derived MSCs on diabetic kidneys could be due to suppression of inflammatory response and oxidative stress (113).

More recent investigations have expanded mechanism of action studies to examine the effects of either allogeneic (114) or syngeneic (115) MSC administration on glomerular podocyte injury. Human adipose-derived MSCs are able to prevent high glucose induced podocytic apoptosis and injury mainly by secreting soluble EGF (114). Administration of bone marrow-derived MSCs via the left renal artery of diabetic rats prevented the development of albuminuria and the loss of podocytes and resulted in a suppressed increase in kidney weight, kidney to body weight index, creatinine clearance rate, and urinary albumin to creatinine ratio, although the treatment had no effect on blood glucose or body weight levels (115). There was evidence of targeted engraftment of MSCs in the renal tissue as intra-arterial injection led to 20% of glomeruli containing EGFP^+^ cells at 24 h with no evidence of labeled cells in the lung, liver, or spleen. However analysis after 60 days indicated a low level of persistent engraftment as only 3% of glomeruli subsequently retained labeled cells. The authors suggested this low level of engraftment suggested a paracrine mode of action with MSCs exerting their beneficial effects by increasing expression of the podocyte survival factor BMP-7 (115). Modification of the expression of immunomodulators has also been proposed as a possible mechanism for the renoprotective effects of MSCs. In a rodent model of DM and diabetic nephropathy, MSC treatment ameliorated diabetic nephropathy via inhibition of MCP-1 expression by secreting HCP, thus reducing macrophage infiltration and down-regulating Il-1β, IL-6, and TNF-α expression in the renal tissue of diabetic rates (116). Others have found beneficial effects of a targeted treatment of MSC administration, using an ultrasound-targeted microbubble destruction (UTMD) technique. Autologous administration of MSCs to diabetic rats in combination with UTMD reduced blood glucose levels, decreased urinary AERs, prevented renal damage, and enhanced homing of MSCs to damaged renal tissue (117).

These studies indicated that MSC cell therapy of diabetic nephropathy may involve individual or combinatorial effects of various renoprotective processes, e.g., differentiation of cells and regeneration, immune modulation/protection, and/or control of hyperglycemia but the complete derivation of the exact mechanisms of action have yet to be elucidated. As the available animal models only mimic the earlier stages of diabetic kidney disease the impact of MSC transplantation on individuals exhibiting advanced signs of diabetic nephropathy such as nodular glomeruloschlerosis remain unproven. At present there is one actively recruiting clinical study registered at ClinicalTrials.Gov^1^ investigating the safety and efficacy of MSCs in diabetic nephropathy.

### MSC-based therapies for diabetic neuropathy

Mesenchymal stromal cells offer a novel therapeutic option to treat DN. MSCs modulate the central nervous system injured environment and promote repair as they secrete anti-inflammatory, anti-apoptotic molecules, and trophic factors to support axonal growth, immunomodulation, angiogenesis, remyelination, and protection from apoptotic cell death (118). MSCs are known to support angiogenesis, which augments the microcirculation supporting peripheral nerves. This impaired vascular supply has been implicated in the etiology of DN. This pro-angiogenic benefit occurs mostly through a paracrine effect. Transplanted MSCs not only directly differentiate into neurons and endothelial cells on administration, but also secrete a broad range of biologically active factors, generally referred to as the MSC secretome. Secretome analysis of MSCs demonstrates that increased concentrations of FGF, VEGF-A, and nerve growth factor are produced from MSCs (119, 120). These factors are central to nerve and vascular tissue health.

Mesenchymal stromal cells have demonstrated benefit in other inflammatory and ischemic conditions. The therapeutic benefit of MSCs in these instances is now believed to be by short-term (hours to days) paracrine and juxtacrine modulation of immune responses rather than by long-term (days to months) engraftment of the MSCs to the injured site (38). These exists a paucity of data on the effect of MSCs on the treatment of DN at the clinical level, however, pre-clinical data has revealed beneficial effects of MSC administration. In a rodent model of DM, intramuscular injection of MSCs resulted in amelioration of the symptoms of DN. Transplantation of MSCs improved hypoalgesia, delayed motor nerve conduction velocity (MNCV), reduced sciatic nerve blood flow (SNBF), and decreased axonal circularity in diabetic nerves of treated rats. The authors indicated that this was most likely due to a paracrine effect as the MSCs were not incorporated into the tissue structures of recipient animals (118). In an STZ-induced DN mouse model, bone marrow-derived MSCs significantly increased expression of neurotrophic factors and ameliorated nerve conduction velocity in diabetic mice (121). Subsequent improvements in MSC cell preparations to generate anti-inflammatory MSC2 populations resulted in significant improvements in behavioral assays in a mouse mode of painful diabetic peripheral neuropathy (pDPN) and mice treated with these MSC2 cells had decreased serum levels of pro-inflammatory cytokines (38). MSC administration also promoted increased density of sympathetic and parasympathetic nerves in the ventricular myocardium of diabetic rats, increased the ratio of parasympathetic to sympathetic nerve fibers and resulted in the suppression of ventricular arrhythmia inducibility (122).

Clinical investigations have recognized the potential benefits of MSC therapy in the treatment of painful disease such as degenerative disk disease and osteoarthritis (123), however, there is no human data on the benefit of MSC administration for the treatment of DN. The mode of administration, dose, and MSC subpopulation, which is the most efficacious, is yet to be determined. Nonetheless, in light of the exciting preclinical evidence of the benefit of MSCs on immunomodulation, angiogenesis, and neurogenesis coupled with the emerging evidence on the glucose lowering effect of MSC therapy, this cell-based treatment may synergistically improve nerve function and alleviate the symptoms and clinical consequences of DN. This will potentially reduce the burden of neuropathic ulceration, pain, and impaired quality of life associated with DN. MSC treatment offers potential benefit in humans with DN, which is currently sub-optimally managed with contemporary treatment strategies.

## MSC-Based Therapies for Other Diabetic Complications

### MSC-based therapies for diabetic wound healing

Effective wound healing is an orchestrated response involving angiogenesis, enhanced cellularity, re-epithelialization, and glandularization, and this is indicative of cutaneous regeneration although the specific cell types involved in each event are not yet known (124). Prolonged and incomplete wound healing, caused by compromised angiogenesis, diminished cell recruitment, lack of growth factors, and impaired formation of collagen matrix is another common complication of DM. It has been demonstrated that generally the number of MSC increases considerably in the site of an injury, and that after a vascular trauma a rapid mobilization to the injured site of EPC also takes place. Recent studies suggest that MSC and EPC are a significant proportion of the non-inflammatory cells that migrate to the skin to promote wound healing (125).

A variety of pre-clinical studies have demonstrated a beneficial effect of MSC administration on wound healing and ulceration in diabetic animals albeit with slightly different mechanisms dependant on autologous or allogeneic administration of progenitor cells. Administration of murine diabetic MSCs inhibited angiogenesis but promoted wound healing in diabetic mice (126) while healthy murine bone marrow-derived MSCs or their conditioned medium were sufficient to promote would healing through differentiation and angiogenesis (127, 128). Bone marrow-derived MSC have also been shown to improve wound healing in diabetic rats following topical or systemic administration (129). After IV administration of MSCs, diabetic wounds showed significantly increased collagen levels in addition to significantly increased levels of growth factors (e.g., EGF, PDGF, and VEGF) that resulted in repair of injured tissue and successful would healing by means of increased secretion of chemokines and increased neovascularization (129). MSCs have also been identified as having direct effects on wound healing through differentiation and regeneration of damaged tissue (127, 130). MSCs promoted angiogenesis through enhanced capillary density and the progenitor cells themselves settled in the newly formed dermis (127, 128, 130). The therapeutic efficacy of bone marrow-derived MSCs to heal fascial and cutaneous wounds was investigated in Sprague-Dawley rats (131). Systemic administration of single or multiple doses of syngeneic MSCs resulted in a significant increase in wound bursting strength 7 and 14 days post wounding. Local administration of MSCs also promoted wound healing and the authors found that allogeneic MSCs were as efficient as syngeneic MSCs in promoting wound healing (131). Local administration of MSCs pre-stimulated with EGF restored blood flow and vasculogenesis in an ischemic hind-limb of type 2 diabetic db/db mice (132). Interestingly, the intradermal injection of adipose-derived MSCs to a dorsal rodent wound improved would healing when compared to control rats but this occurred without a significant effect on angiogenesis or fibroblast accumulation, which lead the authors to suggest the beneficial effect observed was most probably due to the secretion of protective and anti-apoptotic factors such as VEGF and HGF (133).

As indicated above, MSCs are known to promote angiogenesis with improved cutaneous wound healing and biomaterials may increase viability of cells and thus enhance therapeutic efficacy. We previously hypothesized that topically applied allogeneic MSCs wound improve wound healing by augmenting angiogenesis and tested this hypothesis in an alloxan-induced diabetic rabbit ear ulcer model (134) where allogeneic MSCs were seeded in a collagen scaffold and were then applied to a full thickness cutaneous wound on the diabetic rabbit ear. Three doses of MSCs were analyzed and percentage wound closure and angiogenesis were assessed 1 week following cell treatment. Topical application of 1 × 10^6^ MSCs showed increased percentage wound closure compared to lower doses and resulted in increased angiogenesis when compared to untreated wounds. Following on from this study, we are currently investigating the use of a novel antibody (ORB1) that can be used to FACS-isolate ORB1^+^ MSC from human bone marrow with enhanced purity ratios. In this model, we are investigating the effect of xenotransplantation of human MSCs in a collagen matrix to the same alloxan-induced diabetic rabbit ear ulcer model.

Clinical investigations are already underway investigating the effect of local administration of MSCs to chronic non-healing wounds and there is significant interest in the clinical translation of MSC-based therapies to promote dermal regeneration. MSCs are readily available from commercial allogeneic sources or as autologous sources that can be harvested at the point of care from various tissues. Initial clinical studies centered on the beneficial effects of bone marrow aspirate (BMA) or MSC administration on chronic wounds but in the case of diabetes they were often single case reports. Locally applied BMA to a chronic neuro-ischemic wound in a T2DM patient resulted in restored angiogenesis and improved wound healing (135). The combination of the application of bone marrow-derived MSCs with a fibroblast collagen membrane resulted in complete wound closure of a 25-year open wound foot ulcer within 4 weeks (136). Topical administration of autologous bone marrow MSCs to a diabetic patient with an ischemia-induced foot ulcer also demonstrated positive effects on wound healing (137).

In the light of the extensive preclinical data demonstrating the beneficial effects of MSCs in the promotion of dermal wound healing, the clinical translation of these cells remains fairly limited (with small patient populations), but initial clinical studies are just as promising. Autologous culture-expanded bone marrow-derived MSCs applied via a fibrin spray to 13 patients with acute and chronic wounds demonstrated that MSCs migrated into the wound and appeared to stimulate elastin expression resulting in the synthesis of a dermal matrix with improved ECM composition. Acute wounds healed within 8 weeks and chronic year long lower extremity wounds significantly decreased or healed within 16–20 weeks (130). Autologous bone marrow-derived MSCs seeded on a collagen sponge were effective to facilitate the closure of ulcerated wounds (138). This study included 20 patients with non-healing wounds of various etiologies and the authors reported nearly complete healing in 18 patients with vascular regeneration of native tissue evident by immunohistochemical analysis. The effects of the administration of autologous bone marrow-derived MSCs to non-healing lower extremity wounds was investigated in a level one randomized controlled trial of 24 patients whereby MSC therapy significantly reduced wound size and improved clinical parameters (139). All of these studies have confirmed the safe therapeutic benefit of MSCs in the clinical setting of wound healing. As regards to larger clinical studies of wound healing in DM patients only four studies are registered at ClinicalTrials.Gov^1^ one has unknown status and one is not yet recruiting. Of the two completed studies, one had results available. In this clinical study, bone marrow-derived stem cells were applied to treat ischemia-induced chronic foot ulcers in 22 diabetic patients and 18 patients exhibited wound healing after 45 weeks, with improvements in microvascularization detected in some but not all patients (140). Thus, while a variety of pre-clinical and clinical studies have demonstrated very beneficial effects of MSCs in relation to wound healing in diabetic and other patients, larger phase one and two studies are needed to confirm the value of MSCs in wound therapy.

### MSC-based therapies for diabetic cardiomyopathy

Both types of DM increase the progression of atherosclerosis and the development of macrovascular complications, with clinical manifestations such as CAD, peripheral artery disease (PAD), and stroke, and these patients have a two to fourfold increased risk of fatal MI (141, 142). Development of ventricular dysfunction in patients with DM in the absence of CAD, valvular heart disease or hypertension is defined as diabetic cardiomyopathy (DC) (143). DC caused by hyperglycemia causes changes in the diabetic myocardium such as hypertrophy, apoptosis of cardiomyocytes, and abnormal myocardial matrix deposition. Specifically in DC, there are changes in the activity of matrix metalloproteases MMP-2 and MMP-9. Reduced MMP-2 activity results in increased collagen accumulation and increased activity of proapoptotic MMP-9 and subsequent cell apoptosis, capillary density reduction, and poor myocardial perfusion. Other pathological consequences include microcirculatory defects, and interstitial fibrosis (143–145).

The application of MSCs in the treatment of DC (in addition to other CVDs) has received much attention in preclinical and clinical environs in recent years and MSCs do offer promising treatments due to their direct differentiation to cardiomyocytes but also due to the secretion of potent trophic and paracrine mediators, capable of inducing cardioregeneration and cardioprotection. This was elegantly demonstrated in studies of rats with type 1 DM. Intravenous administration (into the femoral vein) of bone marrow-derived MSCs to rats with DC resulted in improved cardiac function of the treated animals through increased angiogenesis and attenuated cardiac remodeling. Transplanted MSCs differentiated into cardiomyocytes and improved angiogenesis and myogenesis. MMP2 activity increased, MMP-9 activity decreased, and there was reduced collagen content in the diabetic myocardium (146). Intramyocardial transplantation of MSC was found to have a protective effect on the diabetic myocardium and DC, and anoxic pre-conditioning (AP) of MSCs was found to enhance this protective effect. In a rodent model of DC, 2 weeks after MSC administration, MSC and AP-MSC groups increased the fractional shortening of the diabetic heart. AP-MSC groups increased myocardial capillary density, attenuated myocardial fibrosis, and inhibited cardiac cell apoptosis (147).

Pre-conditioning of diabetic MSCs with medium from cardiomyocytes exposed to oxidative stress and high glucose (HG/H-CCM medium) also had a beneficial effect on cardiac tissue regeneration. In a mouse model of DM, autologous MSCs preconditioned with HG/H-CCM exhibited upregulated gene expression of angiogenic and cardiac markers. When these cells were implanted by intramyocardial injection into the hearts of STZ-induced diabetic mice (approximately 0.1 × 10^6^ MSC/animal), cardiac function was markedly improved. Pre-conditioned MSCs demonstrated increased homing ability, increased expression of angiogenic and cardiac markers and paracrine mediators, such as IGF-1, HGH, SDF-1, and FGF-2. Four weeks after transplantation reduced fibrosis, apoptosis, and increased angiogenesis was observed in the mouse diabetic hearts (148). This effect was not seen, however, in a mouse model of obesity-induced DC. IV administration of allogeneic bone marrow-derived MSCs with a single dose of 0.5 × 10^6^ or three consecutive monthly doses of 0.5 × 10^6^ MSCs did not result in improved cardiac function when assessed 4 months later but rather has a neutral effect on DC. The observed effects may be as a result of the route, time, and dose used but possibly also to issues with efficient homing and engraftment of the tail vein administered cells (149).

More positive results were seen in a rodent model of dilated cardiomyopathy (DCM). MSC transplantation (5 × 10^6^ cells by injection into the rat myocardium) resulted in induction of myogenesis and angiogenesis and secretion of large amounts of angiogenic and anti-apoptotic factors (VEGF, HGF, adrenomedulin (AM), and IGF-1). A comparison of conditioned medium from MSC versus mononuclear cells MNC revealed MSCs secreted fourfold more VEGF than MNCs. Transplanted MSCs differentiated into cardiomyocytes, vascular endothelial cells, and smooth muscle cells and there was improvement in cardiac function, inhibition of ventricular remodeling, and a decrease in collagen volume in the myocardium with a reduction in myocardial fibrosis when compared to untreated tissue (150).

MSCs administration can also result in improvements in cardiac function through secretion of paracrine mediators, such as Bcl-2, HSP20, and hypoxia-regulated heme oxygenase-1, hypoxic Akt-regulated stem cell factor, VEGF, HGF among others (151). Recent evidence strongly suggests these factors affect remodeling, regeneration, and neovascularization leading to improvements in myocardium contractility and viability (54). Release of trophic mediators by MSCs had also been suggested. Intramuscular delivery of MSCs resulted in improved ventricular function in a hamster heart failure model with enhancement of the density of capillaries and myocytes and a reduction in apoptosis and fibrosis (152).

Based on promising pre-clinical studies, clinical trials have been initiated investigating the effects of MSC transplantation on MI and ischemic cardiomyopathy. Intracoronary administration of autologous BM MSCs to 34 patients with subacute MI demonstrated improvements in perfusion defects and left ventricular chamber size and ejection fraction (EF) 3 months after administration (153). Similarly, 3 months after intravenous administration of allogeneic BM MSCs to patients with acute MI, beneficial effects such as improved pulmonary function, left ventricular EF, and reduced ventricular tachycardia were observed in the hMSC treatment groups. This study also provided important safety data for the administration of allogeneic MSCs to human populations (154). Furthermore, with intramyocardial injection of BM MSCs beneficial effects have been noted in clinical trials of ischemic cardiomyopathy. Three months after treatment, autologous bone marrow MSCs and MNCs cell therapy groups exhibited functional recovery in scarred myocardium and reverse modeling of the LV chamber though the number of patients in this study was low (*n* = 8) (155). A variety of other clinical trials are underway investigating effects of MSCs on heart disease (156) and various possible mechanisms of MSC-mediated cardiac improvement have been suggested including transdifferentiation, paracrine signaling, somatic reprograming, and direct electrophysical coupling (157) but precise delineation of the functional consequences of MSC administration remains to be elucidated.

### MSC-based therapies for diabetic bone fractures

Research over the past few decades has accumulated to indicate that diabetes adversely affects bone quantity and/or quality, and that these skeletal changes, in combination with the microangiopathic complications of diabetes, may increase the risk of bone fracture (158). A systematic review of six patient studies has identified that indeed there is a six to sevenfold increased risk of hip fracture in T1DM rather than non-diabetic individuals with increased risk in other sites too such as spine, proximal humerus and foot (159, 160). With T2DM inconsistent results have been reported in relation to the disease and fracture risk however there are more consistent reports in the literature in relation to the incidence of hip fracture. The risk of hip fracture was increased by 18% in men and 11% in women with DM and in particular T2DM in a large scale Canadian study of diabetic and age-matched non-diabetic controls (161). Furthermore in a recent large scale retrospective analysis of 16 independent studies, there was significant positive associations between the incidence of any non-vertebral fracture, hip fractures, and foot fracture and T2DM (162).

Proposed mechanisms underlying the increased fracture risk in DM include changes in bone mass as a result of hyperglycemia with low bone turnover observed in patients with T1DM and T2DM (158) and changes in bone quality. Rodent models and initial clinical studies suggest DM results in a general weakening or fragility of bone structure that does not necessarily significantly affect elasticity, strength, or fracture toughness (163–165) but may be due to accumulation of advanced glycation end-products (AGEs) within bone collagen (166). Complications of T2DM including retinopathy and autonomic dysfunction may contribute to bone fracture by increasing fall risk (167).

Bone regeneration is reliant on a close spatial and temporal connection between blood vessels and bone cells thus angiogenesis plays a crucial role in skeletal development and bone fracture repair (168). The most critical factor in fracture union is blood supply to the fracture site, which is usually impaired in patients with DM (169) thus diabetic patients are more prone to non-union bone fractures. Current protocols to repair bone defects include autologous or allogeneic bone grafts or implants (e.g., polymeric or metallic), however, there are problems with the above approaches including lack of adequate supply, disease transmission, rejection, cost, and the inability to integrate with the surrounding host tissue. For these reasons combination therapies of stem cells in scaffolds with various growth factors to promote angiogenesis and osteogenesis are being investigated as a tool for bone regeneration (168). Bone healing assisted by MSC can be a powerful clinical tool for bone regeneration because of their ability to differentiate directly into osteoblasts (170, 171) in addition to secretion of pro-angiogenic factors such as VEGF.

To this end, combined delivery of pro-osteogenic factor BMP-4 and pro-angiogenic VEGF in a poly (lactic-co-glycolic acid) scaffold with human bone marrow-derived MSCs resulted in a significant increase in the quantity of regenerated bone compared with single or dual combinations of these factors, when measured by DXA, X-ray and histomorphometric analysis (172). When MSC-titanium implant complex were implanted in the right tibia of type 2 diabetic rats, bone volume ratio and trabecular thickness increased significantly and trabecular separation decreased significantly after 8 weeks when the MSC-implant complexes were compared to titanium implants alone. Histological analysis indicated a greater amount of bone tissue formed around the MSC-implant complexes with a higher bone implant contact rate (BIC) than titanium alone (173). In a rat model of DM, even the use of conditioned medium from MSCs was sufficient to promote fracture healing. MSC derived conditioned medium has been shown to contain significantly higher levels of angiogenic factors such as VEGF and IL-6. When gelatine sponges were soaked in conditioned medium from MSCs and were then implanted into fibular defects in a rodent model of DM, the conditioned medium was sufficient to enhance bone growth and fracture healing compared to control media (169).

The use of allogeneic MSCs to repair non-diabetic bone defects in other rodent or canine models has also been investigated. In rats with a femoral segmental defect, BMP-2-engineered allogeneic MSCs repaired bone defects to the same degree as in rats treated with BMP-2-engineered autologous MSCs. It was also demonstrated that allogeneic gene-transferred MSCs are directly involved in bone repair, in addition to acting as gene deliverers (174). Use of allogeneic MSCs loaded on hydroxyapatite–tricalcium phosphate implants enhanced the repair of a critical-sized segmental defect in dog femurs without the use of immunosuppressive therapy (175). In this case, no adverse immune response was detected. Furthermore, the lack of detected immunogenicity of allogeneic MSCs in these orthopedic studies is an advantage for the clinical application of pre-constructed tissue-engineered bone.

There is a lack of clinical data on the use of MSCs in the treatment of diabetic non-union bone fractures, however, use of the cells has been investigated for the treatment of other bone diseases albeit for small numbers of patients. In a study of 6 children suffering from Osteogenesis imperfect (OI), systemic administration of allogeneic MSCs resulted in acceleration of bone growth for five of the six children (176). In children with hypophosphatasia, a rare heritable metabolic bone disease, bone marrow transplants resulted in marked improved in clinical symptoms, which continued several years after transplant administration although reported studies were single case reports (177, 178).

### MSCs and the REDDSTAR project

**Figure 1:**
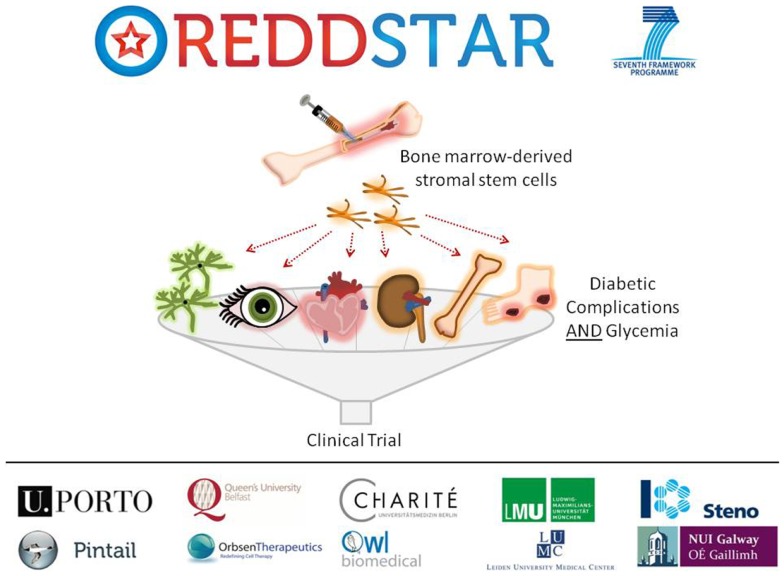
**The REDDSTAR project (www.reddstar.eu)**. The REDDSTAR (Repair of Diabetic Damage by Stromal Cell Administration) consortium is a multinational collaboration involving academic groups in the EU with expertise in the vascular damage resulting from complications of DM. The aim of the REDDSTAR consortium of diabetes specialists, regenerative medicine researchers, biotech industrialists, and clinicians is to significantly impact the management and treatment of the complications of DM. REDDSTAR is novel in its reach across the control of blood glucose and the improvement of a range of six serious diabetic tissue complications: retinopathy, cardiomyopathy, nephropathy, wound healing, neuropathy, and bone fracture repair.

Poor control of blood glucose in DM levels leads to a number of diabetic complications as indicated previously, including: retinopathy, nephropathy, cardiomyopathy, neuropathy, impaired bone repair, and wound ulceration. At present, there are few therapeutic options available to control initiation and progression of diabetic complications and they continue to present challenging disease management issues for clinicians. We have recently received funding from an EU FP7-HEALTH-2012-INNOVATION-1 Grant for a 3-year multicenter multidisciplinary investigation of the use of MSCs in the treatment of DM (REDDSTAR, Repair of Diabetic Damage by Stromal Cell Administration). The REDDSTAR Project^2^ will comprehensively examine if MSC/Stromal Stem Cells (SSC) can safely control glycemia and alleviate damage caused by six diabetic complications. The REDDSTAR consortium is a network of diabetes specialists, regenerative medicine researchers, biotech industrialists, and clinicians, supported by an experienced project management team (Figure 1).

REDDSTAR partner Orbsen Therapeutics has identified a novel antibody (ORB1) that can be used to prospectively isolate ORB1^+^ SSC from human bone marrow with enhanced purity ratios. This new SSC platform technology is a radical improvement in terms of cell purity and compliance with upcoming regulations and REDDSTAR will investigate the efficacy and mechanism of action of this second-generation SSC in six major diabetic complications. The first 18 months of the project will involve investigating the pre-clinical safety and efficiency of SSC in resolving the six complications arising from diabetes. The impact of SSC upon blood glucose levels will also be tested. The second 18 months of the project will involve examining the mechanism of action of how SSC improve diabetic complications. REDDSTAR partners will also submit a clinical trial application to the Danish Medicines Agency to undertake clinical trials on diabetic patients with the complication(s) that yield the best results in phase 1 of the project, thus the REDDSTAR project incorporates both preclinical and clinical development of a novel stem cell therapy within a relatively short timeframe.

## Challenges and Considerations in MSC-Based Therapies and Future Prospects

The application of the use to MSCs to treat microvascular and other common secondary complications of DM has been extensively investigated in pre-clinical animal models in recent years and the majority of reports indicate positive effects on diabetic complications. Despite this, there are significant challenges to be met for the successful clinical translation of these studies from animal model to the patient beside.

The route of administration of MSCs is an important variable. Several modes of cell delivery (e.g., topical, intraocular, and systemic) have been assessed in both pre-clinical and clinical studies in recent times and these studies have illustrated the importance of administration route in the successful outcome of these MSC studies. Systemic delivery is attractive as this may result in benefit for multiple complications and has the potential to improve glycemic control. Although and attractive option, the systemic delivery of MSCs has some barriers such as homing of these cells to tissues of interest with high efficiency and clinically meaningful engraftment. A relatively high number of cells are required for injection due to passive cell entrapment within non-specific tissues (179, 180) and this can potentially lead to unwanted effects and reduced efficacy of transplanted cells. Topical application may be a very relevant alternate strategy for some complications such as diabetic foot ulcers but this is approach can be limited by localized vascular damage as a result of the diabetic milieu at the site of administration.

It has been shown that the duration and degree of cell expansion and culture has an impact on MSC morphology, differentiation, viability, and migratory properties. MSCs not only undergo phenotypic changes in culture and during passage (size, morphology, and cell surface marker expression) (181), but also lose capacity for functional proliferation and differentiation potential (181, 182). In addition, their ability for cytokine production is altered (182). Thus a delicate balance between culture expansion to gain sufficient numbers of MSCs for therapeutic application and long-term culture effects needs to be met. The timing of cell delivery and number of cells delivered are very important however there is still a lack of information as to the optimal cell doses that provide preclinical and clinical efficacy.

Despite numerous studies on the transplantation of MSCs in patient and animal models, insight into the exact mechanisms of action underlying their beneficial effect remains vague. Adequate pre-clinical animal models are required to accurately represent the pathological long-term effects of DM on the host system. There are limitations in the current rodent models of diabetic complications, which tend to show early metabolic and functional disorders but lack marked structural pathology, thus there is uncertainty as to whether investigators are studying mechanisms pertinent to overall pathological damage in humans (93).

An added complication in the scale up of MSC-based technologies is the need to tightly control the microenvironment of the cell. Detailed investigations of how the microenvironment affects the immunosuppressive effects of MSC are still lacking and are required as cell-to-cell contact and soluble factors are thought to be key aspects of MSC-mediated immunosuppression (56). A major challenge is the large scale production of MSCs under GMP conditions and issues of MSC heterogeneity. Furthermore, methods for transportation of MSC-based products without affecting their viability and efficacy are important along with issues related to cryopreservation.

The choice of an autologous or allogeneic approach is an important consideration as the former may be limited by disease-induced cell dysfunction and the latter by an immune response to the transplanted cells. As indicated previously, historical opinions that the immunomodulatory functions of MSCs results in immune privilege for allogeneic MSC transplants are being challenged (79–81) with the recommendation that the anti-donor immune responses elicited by allo-MSCs be studied in more detail.

An additional complication in the clinical translation of MSC therapy is possible malignant transformation and cytogenetic aberrations of MSCs. While most studies have reported no ill effects of MSC transplantation, there are some conflicting reports in the literature. There are some reports of increased tumor formation in animals due to the immune suppressive effect of MSCs especially with allogeneic transplants (183) and *in vitro* observations of sarcoma after culture of murine MSC (184). Other studies have indicated a tumor-suppressive activity of MSC after preactivation with TNF-α (185).

Preclinical animal models are important to test MSC efficacy and mode of action but also are important tools to provide essential information for clinical testing such as safety, toxicity, pharmacokinetics, and pharmacodynamics. The positive pre-clinical data on safety and efficacy generated from a variety of MSC investigations has prompted a huge surge in clinical investigations of MSC therapy. Since the first reported clinical trial of MSCs (186), in excess of 100 clinical trials involving over 3000 human subjects have been performed and no severe adverse events have been reported (187, 188). Thus, even with the reported concerns over possible malignant transformation above, worldwide clinical studies of both autologous and allogeneic MSC administration have confirmed clinical safety and initial efficacy. A search of the ClinicalTrials.Gov website^1^ reveals there are currently 243 open studies of MSC safety and efficacy in the treatment of human diseases, such as acute Graft Versus Host Disease, Acute Lymphoblastic Leukemia, Brain ischemia, and Parkinson’s disease. In relation to DM, there are currently 14 open clinical trials using MSCs to treat T1DM, T2DM, or their associated complications.

## Conclusion

Mesenchymal stromal cells have been highlighted as a promising regenerative therapy due to their multipotency but also due to their paracrine secretion of angiogenic factors, cytokines, and immunomodulatory substances. A variety of pre-clinical and initial clinical studies have indicated that MSCs have potential as a regenerative medicine in diabetes-associated microvascular and secondary diabetic complications. MSC therapies do offer benefits in comparison to other cell-based therapies such as ESC or iPSC in that there are no ethical issues, no sourcing problems, less risk of unrestricted growth, and thus far no adverse effects in clinical trials have been reported. There is increased need for additional *in vitro* and *in vivo* studies to fully describe in detail the mechanisms of MSC-mediated cell therapy, and challenges remain in terms of engraftment, persistence, tissue targeting, and cell manufacture.

## Conflict of Interest Statement

Timothy O’Brien is founder, director, and equity holder in Orbsen Therapeutics and has researcher grants to REMEDI from Medtronic. The other co-authors declare no conflicts of interest.

## Footnotes

^1^http://clinicaltrials.gov/, accessed 30.04.14

^2^http://www.reddstar.eu
